# Maintaining mouth opening to optimize magnetic resonance imaging of the temporomandibular joint: proposal for a new device

**DOI:** 10.1590/0100-3984.2024.0089-en

**Published:** 2025-08-01

**Authors:** Luciana Paula Benício Arcas, Felipe Carlos Dias Arcas, Laís Regiane da Silva Concilio, Marina Amaral

**Affiliations:** 1 Universidade de Taubaté (UNITAU), Taubaté, SP, Brazil

## INTRODUCTION

Temporomandibular disorders (TMDs) encompass two major groups of conditions: those that affect the joint and those that affect the muscles. The maximum mouth opening is one of the parameters employed to assess adequate function and is a diagnostic indicator of a TMD^**(1)**^. In joint-related TMDs, limited mouth opening can be associated with disc displacement without reduction, whereas it can be due to pain or fear of pain in muscle-related TMDs.

The main complementary examination for the evaluation of joint disorders is magnetic resonance imaging (MRI), which is the gold-standard imaging examination for the evaluation of TMDs. Joint dynamics should be evaluated at different degrees of mouth opening^**(2,3)**^. However, keeping the mouth of a patient sufficiently open during the MRI examination can be challenging, and inappropriate management can hinder the diagnosis.

## STATE OF THE TECHNIQUE

In joint-related TMDs, internal disorders of the temporomandibular joint (TMJ) are classified according to the Diagnostic Criteria for Temporomandibular Disorders as follows^**(4)**^: disc displacement with reduction (DD+R); DD+R with intermittent locking; disc displacement without reduction (DDwoR); DDwoR with limited mouth opening (DDwoR+L); and DDwoR without limited mouth opening. Although DD+R is the most common disc disorder of the TMJ, DDwoR+L has been associated with major, irreversible damage to the TMJ.

In the TMJ, DDwoR+L can trigger pain and degenerative processes. Degenerative processes have been shown to be 60% more common in young patients with DDwoR+L than in those without limited mouth opening^**(5)**^. In children, the increased risk of degeneration is even more critical, making early diagnosis and treatment imperative^**(6)**^.

Of treatment-seeking patients, approximately 65% have joint-related and muscle-related conditions, 5% have only joint-related TMD, 13% have only muscle-related TMD, and the remainder have another condition^**(7)**^. For evaluating TMJs, MRI is the imaging examination of choice and, according to the Diagnostic Criteria for Temporomandibular Disorders, should be performed to confirm clinical diagnoses. Soft and hard joint tissues, as well as the dynamics of joint movement, can be evaluated by MRI in different weightings (T1, proton density, and T2 with fat suppression), with the mouth closed, half-open, or open^**(8)**^. However, in several situations, such as myalgia, because of pain or fear of pain, the patient often does not open their mouth correctly, making it difficult to arrive at an accurate diagnosis.

The examination is currently performed with devices of pre-established sizes or disposable syringes to keep the mouth open during the examination. However, in addition to causing patient discomfort, the lack of standardization can compromise the quality of the results, making it impossible to reproduce them, which would be of great value for follow-up, and generating uncertainty regarding the maximum mouth opening during the procedure.

### Proposed device

**Figure 1:**
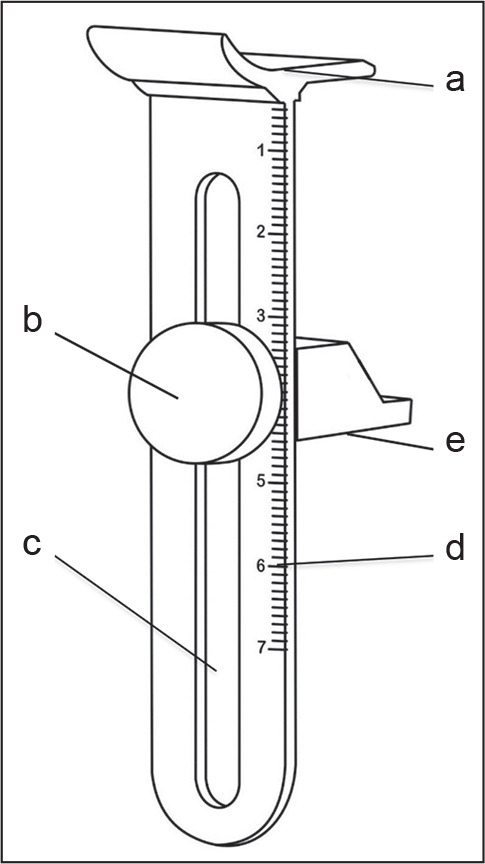
Mouth opening maintenance device. Front view with identification of the constituent elements.

**Figure 2:**
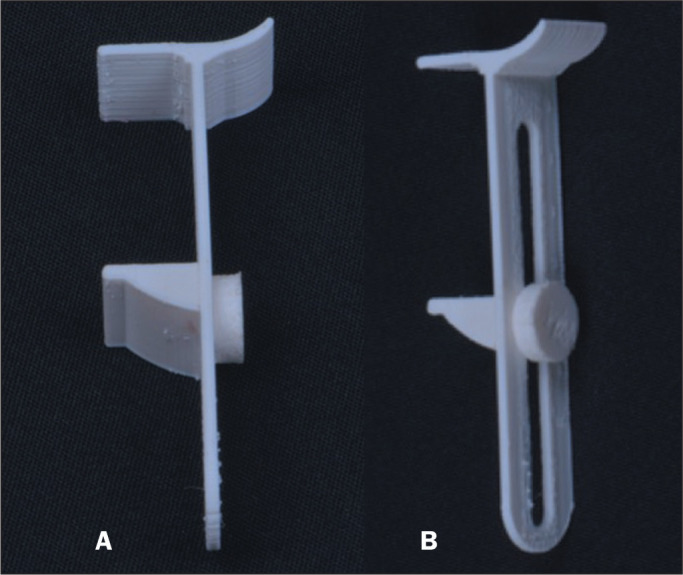
Photo of the prototype of the device three-dimensional printed in resin. A: Side view. B: View of the front, on which millimeter markings will be printed to record the measurements.

**Figure 3:**
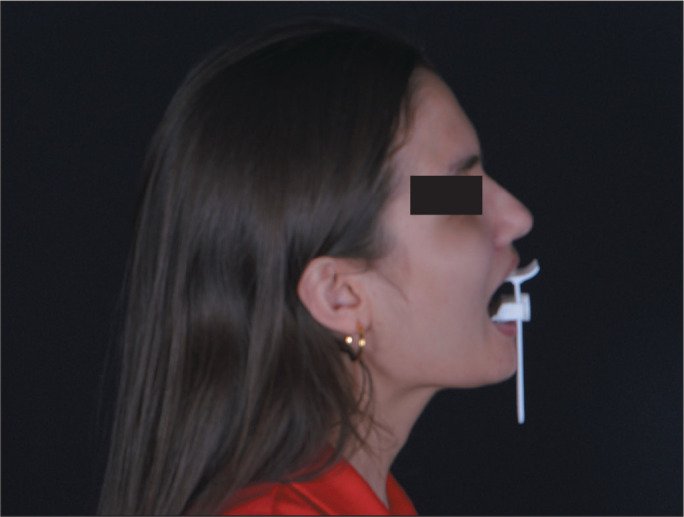
Illustration of how to use the device on a patient.

Figure 1 illustrates the proposed device, consisting of the body (a) with a tightening knob (b) that moves through a channel (c) to be adjusted to the mouth opening selected by the professional, guided by a lateral millimeter ruler (d) and a support for mandibular opening inserted between the teeth (e). This device can be used as a sliding caliper to measure the range of mouth opening with the lateral ruler, and these characteristics make it easy to use without the need for specific training. Figure 2 shows a prototype of the device, three-dimensional printed in resin, for an initial evaluation. Figure 3 shows how the proposed device is used in order to measure mouth opening and keep the mouth open during an MRI examination.

a, body; b, tightening knob; c, sliding channel; d, millimeter ruler; e, dental support for mandibular opening.

The device can be used by the specialist from the initial consultation onward, to determine the values for maximum mouth opening, maximum pain-free opening, and maximum assisted opening and enter them into the clinical record, in which the measurements are established together with the patient. For the MRI examination, the device can be locked at the maximum opening mark. The patient can then take the pre-adjusted device to use at the time of the MRI, or the technician can position the device at maximum opening and indicate in the report the degree of mouth opening at which the examination was performed. The advantages go beyond adequate communication between technicians and clinicians, given that the possibility of personalizing the adjustment will also promote greater patient comfort.

## CONCLUSION

Because DDwoR+L can cause irreversible damage to the TMJ, rapid, accurate differential diagnosis of the cause of limitation, between mechanical interposition of the disc and myofascial pain, should be performed in order to determine the proper treatment.

The device presented here can, in a simple way, solve a recurring difficulty in MRI examinations of the TMJ and facilitate diagnosis by the specialist.
